# Unexpected diagnosis: a case of incidental congenital diaphragmatic hernia

**DOI:** 10.1093/omcr/omae193

**Published:** 2025-03-20

**Authors:** Daniela Saenz, Juan P Cóbar, Victor H Argueta, Ricardo A Caravantes

**Affiliations:** Department of Medical Research, Universidad Francisco Marroquín, 6ta calle final zona 10, Universidad Francisco Marroquin, Guatemala City, 01010, Guatemala; Department of Medical Research, Universidad Francisco Marroquín, 6ta calle final zona 10, Universidad Francisco Marroquin, Guatemala City, 01010, Guatemala; Department of Medical Research, Universidad Francisco Marroquín, 6ta calle final zona 10, Universidad Francisco Marroquin, Guatemala City, 01010, Guatemala; Department of Medical Research, Universidad Francisco Marroquín, 6ta calle final zona 10, Universidad Francisco Marroquin, Guatemala City, 01010, Guatemala

**Keywords:** congenital diaphragmatic hernia, respiratory distress, pulmonary hypoplasia, surgical repair

## Abstract

Congenital diaphragmatic hernias (CHD) are rare anomalies resulting from the failure of the diaphragm to form. Bochdalek hernias are characterized by posterolateral displacement of abdominal organs into the chest. The condition’s clinical presentation is variable and commonly presents with acute respiratory distress after birth. Timely recognition and management are crucial due to the associated morbidity and mortality rates. In the present case, a diaphragmatic hernia was discovered incidentally in a 1-year-old patient following an appendectomy. This case highlights the diverse clinical presentations and diagnostic challenges of Bochdalek hernias, reinforcing the importance of interdisciplinary collaboration for effective patient care.

## Introduction

Congenital diaphragmatic hernias (CDH) are anomalies that arise from errors during embryological development [1]. They allow herniation of abdominal organs into the thoracic cavity and are classified as Bochdalek or Morgagni hernias. While both types are rare congenital defects, Bochdalek is the most common [2]. These defects result from a failure of the posterolateral septum transversum to fuse with the pleuroperitoneal membranes during embryogenesis, causing abdominal organs to shift into the thoracic cavity [2].

The clinical presentation of Bochdalek hernias typically includes respiratory distress shortly after birth, but the symptoms can vary depending on the hernia’s size and the organs involved. Diagnosis is often challenging due to this variability, and timely recognition is critical for survival, given the condition’s high morbidity and mortality rates [1].

This case report presents a 1-year-old patient diagnosed incidentally with a diaphragmatic hernia after a surgical procedure. It aims to describe the patient’s presentation, workup, and management, emphasizing an unusual presentation given that there was no history of pulmonary or gastrointestinal manifestations since birth. It is important to mention that the patient was not born in a hospital, did not exhibit any respiratory symptoms that would suggest an underlying abnormality such as the one presented, and had limited pediatric follow-up due to insufficient resources.

## Case report

A 1-year-old female presented to the Department of Pediatric Surgery with abdominal pain and fever. An abdominal ultrasound revealed findings suggestive of appendicitis, leading to an open appendectomy. Intraoperatively, no abnormal findings were noted, and the pathology report confirmed inflammatory changes consistent with appendicitis. However, postoperatively, the patient developed respiratory distress and could not be extubated.

**Figure 1:**
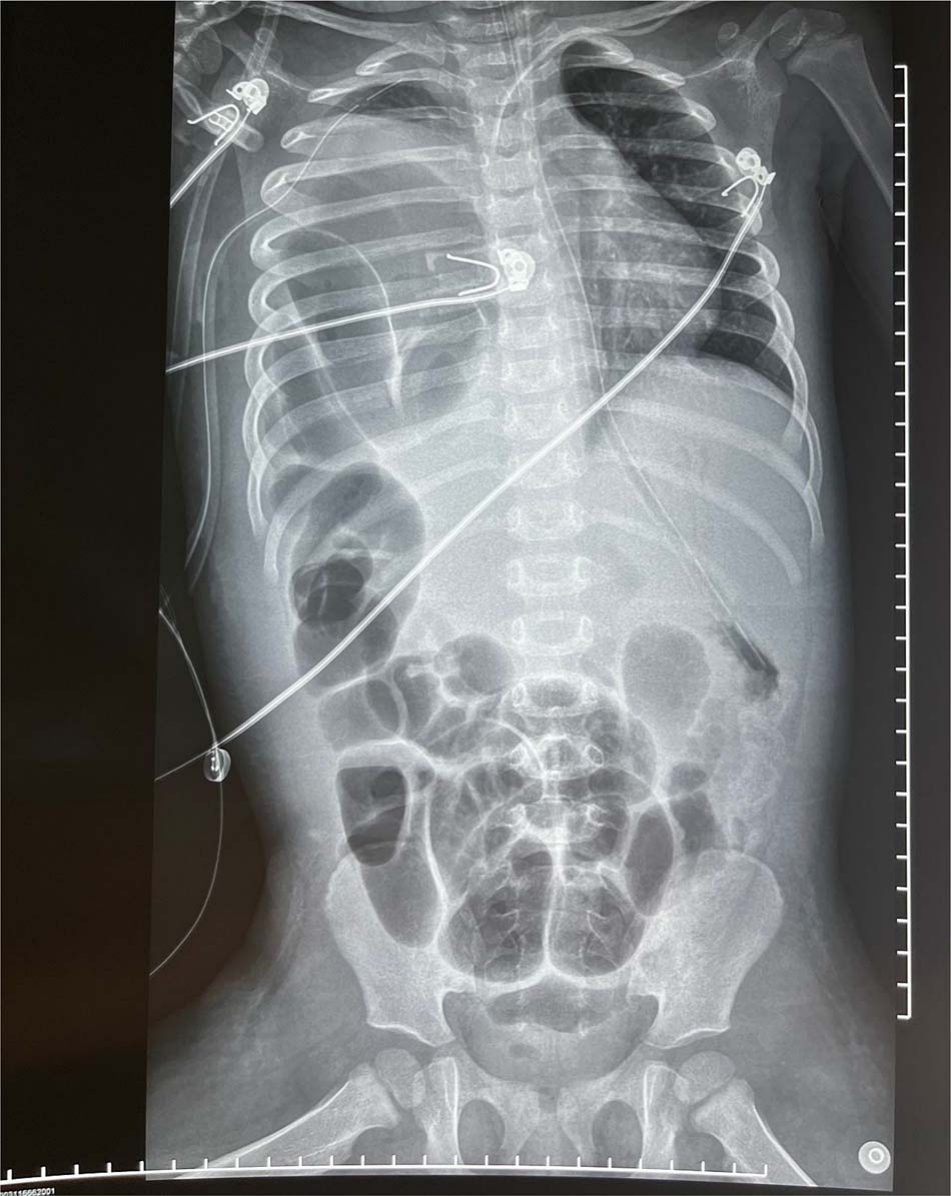
Anteroposterior X-ray showing intestinal loops in the right hemithorax with associated contralateral displacement of the mediastinal structures.

**Figure 2:**
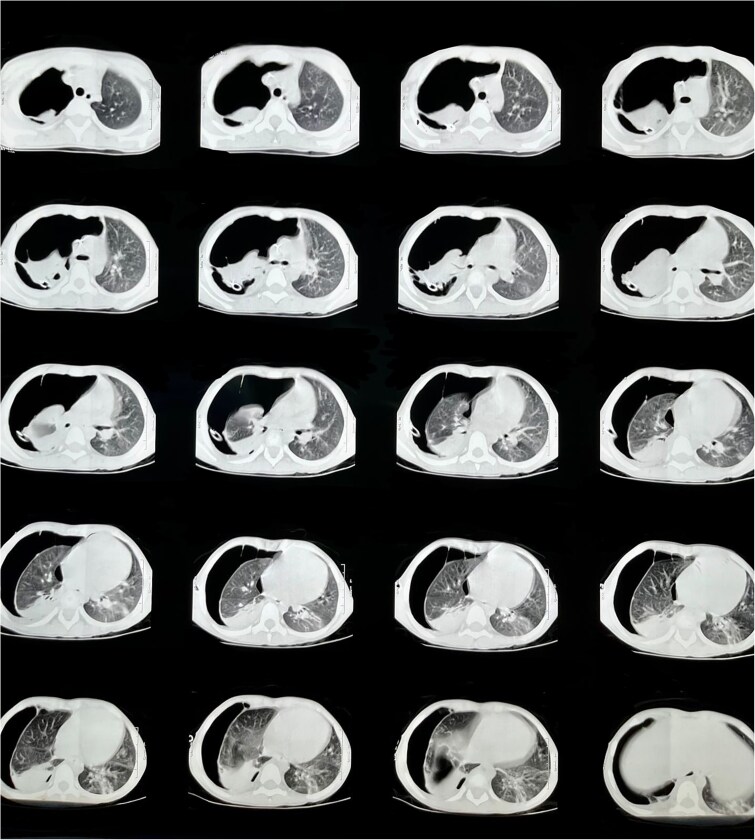
Chest CT scan on lung window showing a hypoplastic right lung.

On physical examination, the patient exhibited tachypnea (RR 65 bpm), tachycardia (HR 148 bpm), and pulse oximetry of 89% on room air, with decreased breath sounds on the right hemithorax and use of accessory muscles of respiration. Initial chest X-ray findings showed a ‘white-out’ appearance of the lung, prompting the placement of a chest tube. A subsequent X-ray revealed intestinal loops in the right hemithorax (Fig. 1). The patient was transferred to a tertiary care facility, where a CT scan confirmed the diagnosis of a diaphragmatic hernia (Fig. 2).

Initially, she was admitted for surgical management; upon evaluation, she presented signs of septic shock and was transferred to the intensive care unit. The clinical presentation of sepsis with an associated diaphragmatic hernia raised suspicion of intestinal perforation.

**Figure 3:**
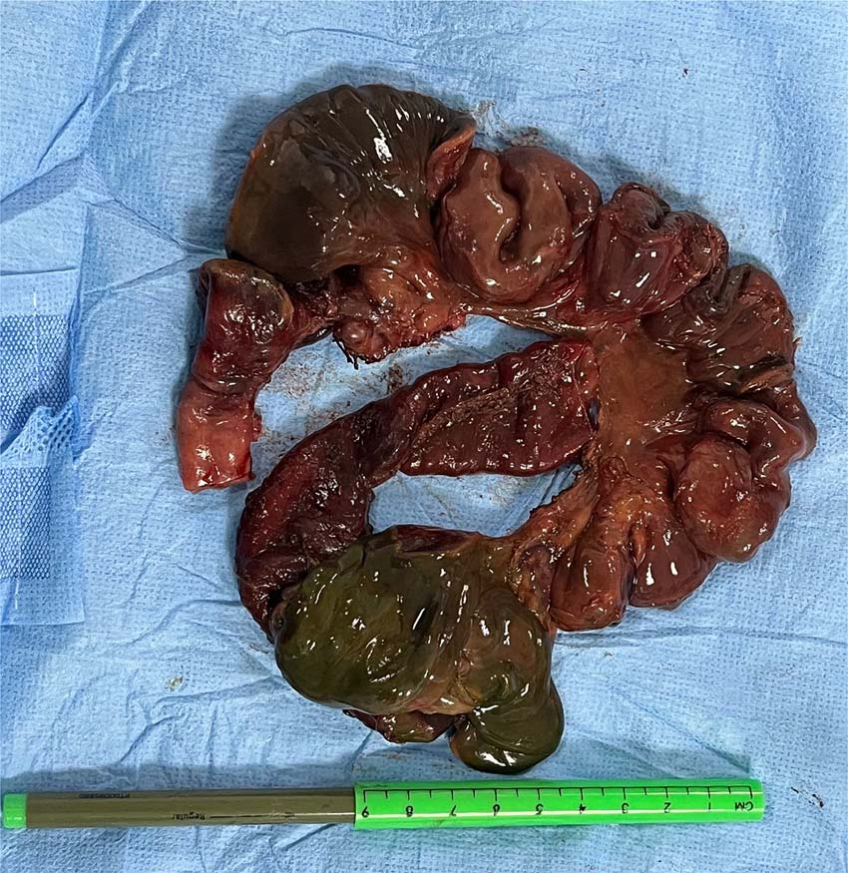
Ileum reduced from the hernia sac and resected with evidence of irreversible ischemic changes.

An exploratory laparotomy was performed for hernia reduction and repair. A right Bochdalek diaphragmatic hernia of 2.5 cm x 2 cm in diameter containing 50 cm of strangulated ischemic distal ileum was identified (Fig. 3). An ileal perforation was visualized, and ileal resection with an ileoileal end-to-end anastomosis was carried out. There were no findings suggestive of intestinal malrotation. A hypoplastic right lung was isolated in the apex of the right hemithorax. The defect was repaired with a primary closure technique. The patient was discharged two weeks after surgery without respiratory or gastrointestinal complications. Notably, she did not develop small bowel syndrome following the ileal resection but continues to attend follow-up visits to monitor for any potential complications.

## Discussion

Bochdalek hernia, a subtype of CDH, is a condition that arises during fetal development and can be life-threatening if not diagnosed and treated promptly [3]. It is reported in 1 in 2200 newborns, although the exact cause remains unclear. Genetic or environmental factors may disrupt mesenchymal cell differentiation, leading to failure of the posterolateral septum transversum to fuse with the pleuroperitoneal membranes [4]. This disruption results in the displacement of abdominal organs into the thoracic cavity [1, 5].

Prenatal ultrasound examinations can usually detect CDH; however, there are instances when it remains undiagnosed until after birth. Newborns with CDH often present with respiratory distress due to pulmonary hypoplasia and compromised ventilation [6]. In cases of late-presenting CDH, such as in this patient, the pulmonary hypoplasia and hypertension tend to be less severe compared to neonatal presentations [7]. Additionally, left-sided hernias may be associated with hypoplastic left heart syndrome, potentially resulting from mediastinal shift and loss of left ventricular muscle mass [8].

If the diagnosis is not made prenatally, postnatal clinical evaluation can reveal respiratory or gastrointestinal distress. Initial diagnostic imaging often involves X-rays, which are highly sensitive and widely available [5]. Confirmation through ultrasound or CT scanning provides a more detailed assessment, and further tests, such as pulmonary function evaluations and echocardiography, can help gauge potential complications [4].

In this case, respiratory distress following an appendectomy led to the incidental discovery of an undiagnosed CDH. This presentation aligns with other reported cases where CDH was detected during unrelated procedures or diagnostic evaluations for gastrointestinal symptoms. The postoperative respiratory distress and failure to extubate prompted further imaging, which revealed the hernia. Similar to other cases, the hernia was complicated by strangulated ischemic bowel and ileal perforation, necessitating immediate surgical intervention. These complications are often life-threatening and emphasize the need for prompt diagnosis and multidisciplinary management.

Management of CDH is complex and requires a team approach due to the physiological derangements involved. Late-presenting CDH, like in this case, can lead to herniation of the stomach, mediastinal shift, tension pneumothorax, or incarceration of abdominal organs. Reducing intrathoracic pressure is critical, and nasogastric tube placement is a simple but effective method, although complications such as kinking can occur [7]. Continuous monitoring of ventilatory status and hemodynamic evaluation is essential, as cardiac compromise can be a consequence of the hernia. Nutritional support and careful fluid management are necessary due to the involvement of the intestines [9].

The definitive surgical treatment involves reducing the hernia and repairing the defect. Primary closure with sutures is preferred in most cases, although large defects may require the use of Gore-Tex patches or abdominal muscle flaps [10, 11]. The timing of surgical repair can be delayed until the patient is medically stabilized, particularly in cases of late-presenting CDH where symptoms are mild [1, 9]. Long-term observation is an effective strategy in patients with minor defects who present minimal or no symptoms.

## Conclusion

This case highlights the unexpected diagnosis of a congenital Bochdalek diaphragmatic hernia in a 1-year-old female following an appendectomy. Postoperative respiratory distress and the inability to extubate led to the discovery of the hernia, complicated by strangulated ischemic ileum and perforation. The case underscores the importance of considering congenital anomalies in pediatric patients with acute symptoms and emphasizes the critical role of imaging in diagnosis. Early identification and timely management are essential to prevent severe complications and ensure favorable outcomes.
